# Uterine Fibroid Diagnosis by Race and Ethnicity in an Integrated Health Care System

**DOI:** 10.1001/jamanetworkopen.2025.5235

**Published:** 2025-04-02

**Authors:** Susanna D. Mitro, Wendy Dyer, Catherine Lee, Ameek Bindra, Lana Wang, Miranda Ritterman Weintraub, Monique M. Hedderson, Eve Zaritsky

**Affiliations:** 1 Division of Research, Kaiser Permanente Northern California, Pleasanton; 2 Department of Obstetrics and Gynecology, Kaiser Permanente Northern California, Oakland; 3 Department of Graduate Medical Education, Oakland Medical Center, Kaiser Permanente Northern California, Oakland

## Abstract

**Importance:**

Uterine fibroids are benign tumors that can cause severe symptoms. Fibroid burden among Asian or Pacific Islander individuals, particularly in specific subgroups, remains largely unexamined.

**Objective:**

To describe fibroid diagnosis rates by race and ethnicity.

**Design, Setting, and Participants:**

This retrospective cohort study (January 2009 to December 2022) used data from electronic health records of the Kaiser Permanente Northern California (KPNC) integrated health care system. Eligible participants included female (sex assigned at birth) KPNC members aged 18 to 54 years with at least 12 months of continuous membership, no prior fibroid diagnosis, and no history of hysterectomy. Data were analyzed January to September 2024.

**Exposure:**

Race and ethnicity was categorized into 9 groups: Black (African American, other Black [ie, any Black race or ethnicity not otherwise specified], and unknown Black race or ethnicity), East Asian (Chinese, Japanese, and Korean), Hispanic (Hispanic or Latino), South Asian (Asian Indian, Bangladeshi, East Indian, Nepali, Sri Lankan, and any South Asian ethnicity not otherwise specified), Southeast Asian (Filipino, Vietnamese, and other Southeast Asian [ie, any Southeast Asian ethnicity not otherwise specified]), White (White or White Middle Eastern), other Asian or Pacific Islander (Native Hawaiian or Pacific Islander, multiethnic Asian, and other or unspecified Asian ethnicity [ie, any Asian ethnicity not otherwise specified]), other races and ethnicities (American Indian or Alaska Native and multiracial), and unknown race or ethnicity.

**Main Outcomes and Measures:**

Incident fibroid diagnosis was identified using diagnostic codes. Poisson regression models were used to calculate incidence rates of fibroid diagnosis by racial and ethnic group, standardized to the 2022 US female population. Incidence rate ratios (IRRs) and 95% CIs compared incidence within each racial and ethnic group with White participants.

**Results:**

A total of 1 917 794 patients were included (median [IQR] percentage, 7% [6%-7%] Black; 5% [5%-6%] East Asian; 22% [21%-23%] Hispanic; 3% [2%-3%] South Asian; 7% [7%-8%] Southeast Asian; 42% [39%-45%] White; 8% [6%-10%] other Asian or Pacific Islander; 2% [2%-2%] of other races and ethnicities; 4% [4%-5%] unknown or missing race and ethnicity), and 84 206 patients (4.4%) received a first fibroid diagnosis during the study period. Compared with White patients, there was a higher rate of fibroid diagnosis among Southeast Asian (IRR, 1.29; 95% CI, 1.26-1.33), East Asian (IRR, 1.47; 95% CI, 1.43-1.51), and South Asian patients (IRR, 1.71; 95% CI, 1.65-1.78). Black (IRR, 3.11; 95% CI, 3.05-3.17) and Hispanic patients (IRR, 1.37; 95% CI, 1.34-1.39) also had elevated rates of fibroid diagnosis. All Asian patients were less likely than other groups to have *ICD-9* or *ICD-10* coded symptoms before diagnosis.

**Conclusions and Relevance:**

In this cohort study of nearly 2 million KPNC patients, South Asian, East Asian, Southeast Asian, Hispanic, and Black patients had higher fibroid diagnosis rates than White patients. If diagnosis rates reflect true variation in disease prevalence, future research should identify sources of these disparities and strategies to reduce them.

## Key Points

**Question:**

Does the rate of fibroid diagnosis vary by race and ethnicity, especially among Asian ethnic groups?

**Findings:**

In this cohort study of 1 917 794 patients, fibroid diagnosis rates were 71% higher for South Asian, 47% higher for East Asian, and 29% higher for Southeast Asian patients compared with non-Hispanic White patients. Diagnosis rates were also elevated among Hispanic and Black patients compared with White patients.

**Meaning:**

This study found that fibroid diagnosis rates were elevated for Asian populations, with important variability among Asian ethnic subgroups, motivating investigation into causes of this ethnic disparity to ensure equitable screening and treatment.

## Teaser

This cohort study assesses differences in rates of uterine fibroid diagnosis by race and ethnicity from 2009 to 2022 in an integrated health care system in California.

## Introduction

Uterine fibroids (leiomyoma) are common noncancerous uterine tumors that can cause severe symptoms including pain, fatigue, and bleeding.^1,2,3^ Up to 70% to 80% of individuals with a uterus in the US may develop fibroids by age 50 years, with 30% to 40% experiencing clinically significant disease.^3^ Treating fibroids is costly; annual expenses for surgeries, medications, and procedures range from $4.1 to $9.4 billion.^4^ Despite high prevalence and high morbidity, the cause of fibroids is poorly understood, limiting prevention efforts.^5^

Research has consistently shown that Black individuals develop fibroids at younger ages, have a larger number of fibroids, and experience more severe symptoms than White individuals.^6,7^ The causes of this racial disparity are not fully understood, but may include racial disparities in chronic psychosocial stress including exposure to racism,^8,9,10^ insufficient vitamin D concentrations,^11,12,13^ and differences in environmental chemical exposures.^14^ Fibroid risk is multifactorial and likely affected by social determinants of health.

Although there is clear evidence of a racial disparity between Black and White individuals in fibroid risk, the risk of fibroids for other minoritized racial and ethnic groups, including Asian individuals (the fastest-growing racial group in the US)^15^ is not well described. Understanding the variation in fibroid risk and disease presentation across racial and ethnic groups is essential to identify modifiable risk factors, counsel high-risk groups, and ensure effective treatment. Using Kaiser Permanente Northern California (KPNC) electronic health records (EHR), we examined the incidence of fibroid diagnosis by race and ethnicity. Importantly, KPNC detailed data allowed us to evaluate fibroid incidence by Asian ethnic subgroups, which is critical in light of growing evidence of variation in health outcomes among these subgroups.^16^

## Methods

This cohort study was approved by the KPNC institutional review board with a waiver of informed consent, because this minimal risk research could not otherwise practicably be carried out, in accordance with 45 CFR § 46. The study adheres to the Strengthening the Reporting of Observational Studies in Epidemiology (STROBE) reporting guideline. KPNC is a large, vertically integrated health care delivery system that provides health care for approximately 4.4 million Northern California residents.^17^ The vast majority of care is provided in a closed system and captured in the EHR. KPNC membership is broadly representative of the regional population.^17^

Using the KPNC EHR, we identified eligible patients, which included KPNC members aged 18 to 54 years who were assigned female at birth and had at least 12 months of continuous membership before study entry (index date) between January 2009 and December 2022. We excluded patients who had a fibroid diagnosis or hysterectomy within the 5 years before the index date. Participants contributed person-time starting on the index date and continuing until reaching age 55 years, undergoing a hysterectomy, receiving a fibroid diagnosis, death, or leaving KPNC membership for longer than 3 months. Participants who left and rejoined KPNC later in the study period could resume contributing person-time after 12 months of continuous enrollment. To calculate annual incidence rates, we created retrospective cohorts for each year and counted all the person-time and fibroid diagnoses occurring in each. Eligible patients could contribute person-time to multiple annual cohorts.

### Race and Ethnicity

We used participants’ self-reported race and ethnicity, supplemented by administrative (non–self-reported) data when no self-reported data were available, to assign participants’ race and ethnicity. We further categorized some races and ethnicities to ensure that each group had an adequate number of participants: Black (African American, other Black race or ethnicity [ie, any Black race or ethnicity not otherwise specified], and unknown Black race or ethnicity), East Asian (Chinese, Japanese, and Korean), Hispanic (Hispanic or Latino), South Asian (Asian Indian, Bangladeshi, East Indian, Nepali, Sri Lankan, and any South Asian ethnicity not otherwise specified), Southeast Asian (Filipino, Vietnamese, and other Southeast Asian [ie, any Southeast Asian ethnicity not otherwise specified]), other Asian or Pacific Islander (Native Hawaiian or Pacific Islander, multiethnic Asian, and other or unspecified Asian ethnicity [ie, any Asian ethnicity not otherwise specified]), White (White or White Middle Eastern), other races and ethnicities (American Indian or Alaska Native and multiracial, which were grouped due to small numbers), and unknown race or ethnicity.

### Fibroid Diagnosis

Incident fibroid diagnosis was defined using the first *International Classification of Diseases, Ninth Revision *(*ICD-9; *code 218x) or *International Statistical Classification of Diseases and Related Health Problems, Tenth Revision *(*ICD-10*; code D25x). Eligible fibroid diagnoses could occur during inpatient, outpatient, emergency department, or virtual care visits.

### Covariates

Participants’ age, year of diagnosis, parity, and body mass index (BMI; calculated as weight in kilograms divided by height in meters squared) were extracted from the EHR. We also extracted symptoms within 2 years before the index diagnosis (excessive, frequent, or irregular bleeding; dysmenorrhea; dyspareunia; lower abdominal or pelvic pain; and urinary incontinence). eTable 1 in Supplement 1 lists *ICD-9* and *ICD-10* codes for each. Finally, we linked participants’ home addresses to census tract–level data to calculate the Neighborhood Deprivation Index, which uses poverty, employment, education, and housing to reflect neighborhood conditions.^18^ Participants missing covariate data were retained in all analyses; we report missing as a category in descriptive tables where applicable.

### Statistical Analysis

We used Poisson regression models to calculate annual and overall incidence rates of fibroid diagnosis within each racial and ethnic group to describe variation among the groups across the study period. We standardized rates to the age distribution of the 2022 US female population to minimize the effect of varying age distributions within each racial and ethnic group. We additionally calculated overall incidence rate ratios (IRRs) to summarize group differences over the study period, comparing the incidence of fibroid diagnosis within each racial and ethnic group to the incidence among White participants. A (2-sided) *P* < .05 was considered statistically significant. The analysis was conducted in SAS 9.4 (SAS Institute) from January to September 2024.

## Results

In total, 1 917 794 patients across annual cohorts (median [IQR] percentage, 7% [6%-7%] Black; 23% [20%-27%] Asian or Pacific Islander [5% (5%-6%) East Asian; 3% (2%-3%) South Asian; 7% (7%-8%) Southeast Asian; 8% (6%-10%) other Asian or Pacific Islander]; 22% [21%-23%] Hispanic; 42% [39%-45%] White; 2% [2%-2%] of other races and ethnicities; 4% [4%-5%] with unknown or missing race and ethnicity) were eligible for a first fibroid diagnosis between 2009 and 2022 (median [IQR], 746 741 [699 995-830 380] participants in each annual cohort), and 84 206 patients (4.4%) received a fibroid diagnosis over the study period (median [IQR], 5833 [5436-6643] participants in each annual cohort). The proportion of patients who had pelvic or abdominal imaging in the 8 weeks before diagnosis was 59% (49 553 of 84 206 patients). Cohort demographic characteristics for 2009 and 2022, as well as the median and proportion of missing data for each characteristic across study years, are shown in Table 1. Patients with a fibroid diagnosis were more likely to be aged 41 to 45 years or 46 to 50 years and less likely to be younger than 30 years, more likely to be Black and less likely to be White, and more likely to be nulliparous and have a BMI of 30 or greater compared with the overall cohort (Table 1). Full demographic characteristics for each year of the study are shown in the eTable 2 in Supplement 1. Reasons that eligible participants were censored in each year are shown in eTable 3 in Supplement 1.

**Table 1.** Median Proportion of Each Cohort With Each Demographic Characteristic, Summarized Across All 14 Annual Cohorts (2009-2022), and Annual Cohort Characteristics Specifically in 2009 and 2022

Characteristic	All 14 cohorts (2009-2022), median (IQR), % (N = 1 917 794)		2009 Cohort, No (%)		2022 Cohort, No (%)	
	Overall^a^	Fibroid diagnosis^a^	Overall (n = 734 955)	Fibroid diagnosis (n = 5846)	Overall (n = 855 111)	Fibroid diagnosis (n =6736)
No. of participants, median (IQR)	746 741 (699 995-830 380)	5833 (5436-6643)	NA	NA	NA	NA
Age, y						
18-29	23 (21-24)	5 (5-5)	213 364 (29)	245 (4)	162 733 (19)	282 (4)
30-34	17 (16-18)	14 (12-15)	99 132 (13)	575 (10)	155 714 (18)	978 (15)
35-39	17 (15-17)	19 (18-20)	102 612 (14)	1043 (18)	151 881 (18)	1316 (20)
40-44	15 (15-15)	24 (23-25)	101 215 (14)	1412 (24)	138 572 (16)	1617 (24)
45-49	15 (14-15)	25 (24-26)	108 965 (15)	1686 (29)	122 078 (14)	1639 (24)
50-54	15 (14-15)	14 (13-14)	109 667 (15)	885 (15)	124 133 (15)	904 (13)
Race and ethnicity						
Black	7 (6-7)	16 (15-17)	55 384 (8)	1069 (18)	51 559 (6)	938 (14)
East Asian^b^	5 (5-6)	7 (6-8)	40 689 (6)	440 (8)	38 734 (5)	434 (6)
Hispanic	22 (21-23)	23 (21-24)	149 108 (20)	1212 (21)	211 841 (25)	1671 (25)
South Asian^c^	3 (2-3)	3 (3-4)	12 763 (2)	103 (2)	25 916 (3)	318 (5)
Southeast Asian^d^	7 (7-8)	8 (8-9)	54 954 (8)	458 (8)	56 152 (7)	477 (7)
Other Asian or Pacific Islander^e^	8 (6-10)	6 (4-8)	37 422 (5)	224 (4)	100 480 (12)	689 (10)
White	42 (39-45)	32 (30-34)	327 079 (45)	2111 (36)	303 691 (36)	1851 (28)
Other^f^	2 (2-2)	2 (2-2)	14 887 (2)	127 (2)	14 405 (2)	117 (2)
Unknown or missing	4 (4-5)	2 (2-2)	42 669 (6)	102 (2)	52 333 (6)	251 (4)
Parity						
0	23 (22-23)	26 (25-26)	119 843 (16)	930 (16)	183 412 (21)	1766 (26)
1	16 (15-16)	16 (15-17)	86 510 (12)	694 (12)	125 138 (15)	1023 (15)
≥2	36 (35-38)	37 (36-40)	192 308 (26)	1781 (30)	281 927 (33)	2254 (33)
Unknown or missing	26 (24-31)	21 (19-25)	336 294 (46)	2441 (42)	264 634 (31)	1693 (25)
Body mass index^g^						
<25.0	35 (32-37)	30 (28-31)	270 313 (37)	1828 (31)	180 085 (21)	1352 (20)
25.0 to <30.0	25 (24-25)	28 (27-28)	179 579 (24)	1632 (28)	154 790 (18)	1353 (20)
30.0 to <35.0	14 (14-14)	17 (17-17)	99 900 (14)	972 (17)	99 608 (12)	954 (14)
≥35	13 (13-14)	16 (15-16)	93 525 (13)	930 (16)	101 193 (12)	949 (14)
Unknown or missing	13 (12-13)	10 (9-10)	91 638 (12)	484 (8)	319 435 (37)	2128 (32)
Neighborhood Deprivation Index quartile						
1: Least deprived	19 (17-22)	18 (17-22)	112 246 (15)	894 (15)	198 198 (23)	1613 (24)
2	32 (29-32)	31 (29-32)	233 485 (32)	1780 (30)	239 245 (28)	1878 (28)
3	28 (27-28)	28 (28-30)	219 593 (30)	1794 (31)	234 338 (27)	1857 (28)
4: Most deprived	21 (21-22)	21 (21-23)	166 805 (23)	1373 (23)	182 576 (21)	1384 (21)

Abbreviation: NA, not applicable.

^a^ The total number of patients across all 14 annual cohorts was 1 917 794. The total number with a fibroid diagnosis across all 14 annual cohorts was 84 206. eTable 2 in Supplement 1 contains the full characteristics of the eligible population in each annual cohort.

^b^ East Asian included Chinese, Japanese, and Korean.

^c^ South Asian included Asian Indian, Bangladeshi, East Indian, Nepali, Sri Lankan, and any South Asian ethnicity not otherwise specified.

^d^ Southeast Asian included Filipino, Vietnamese, and other Southeast Asian (ie, any Southeast Asian ethnicity not otherwise specified).

^e^ Other Asian or Pacific Islander includes Native Hawaiian or Pacific Islander, multiethnic Asian, and other or unspecified Asian (ie, any other Asian ethnicity not otherwise specified).

^f^ Other includes American Indian or Alaska Native and multiracial. Numbers are not standardized by age.

^g^ Body mass index was calculated as weight in kilograms divided by height in meters squared.

**Figure 1.**
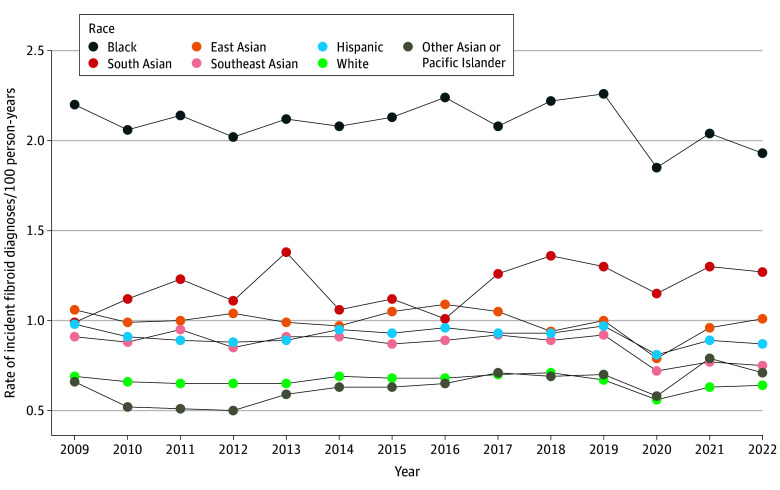
Annual Incidence of Fibroid Diagnosis Per 100 Person-Years by Race and Ethnicity Incidence rates are age-standardized to the 2022 US female population. Plotted values are listed with confidence intervals in eTable 5 in Supplement 1. East Asian included Chinese, Japanese, and Korean. South Asian included Asian Indian, Bangladeshi, East Indian, Nepali, Sri Lankan, and any South Asian ethnicity not otherwise specified. Southeast Asian included Filipino, Vietnamese, and other Southeast Asian (ie, any Southeast Asian ethnicity not otherwise specified). Other Asian or Pacific Islander includes Native Hawaiian or Pacific Islander, multiethnic Asian, and other or unspecified Asian (ie, any other Asian ethnicity not otherwise specified).

**Figure 2.**
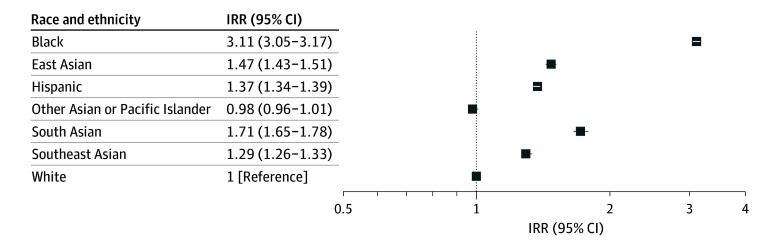
Incidence Rate Ratios (IRRs) and 95% CIs for Fibroid Diagnosis Compared With White Patients Incidence rates reflect the full study period (2009-2022) and are age-standardized to the 2022 US female population. East Asian included Chinese, Japanese, and Korean. South Asian included Asian Indian, Bangladeshi, East Indian, Nepali, Sri Lankan, and any South Asian ethnicity not otherwise specified. Southeast Asian included Filipino, Vietnamese, and other Southeast Asian (ie, any Southeast Asian ethnicity not otherwise specified). Other Asian or Pacific Islander includes Native Hawaiian or Pacific Islander, multiethnic Asian, and other or unspecified Asian (ie, any other Asian ethnicity not otherwise specified).

Fibroid diagnosis rates were generally consistent over the study period for most racial and ethnic groups (Figure 1). Diagnosis rates for South Asian patients increased in general over the study period. Compared with 2009, rates of fibroid diagnosis were significantly lower in 2020 for White, Black, East Asian, and Hispanic patients (eTable 4 in Supplement 1). The age-standardized incidence of fibroid diagnosis per year among White patients ranged from 0.56 (95% CI, 0.53-0.58) to 0.71 (95% CI, 0.68-0.74) diagnoses per 100 person-years between 2009 to 2022 (eTable 5 in Supplement 1). Compared with White patients, Black patients had more than 3 times greater age-standardized incidence of fibroid diagnosis (incidence rate range, 1.85 [95% CI, 1.72-1.97] to 2.26 [95% CI, 2.12-2.40] diagnoses per 100 person-years; IRR; 3.11; 95% CI, 3.05-3.17) (Figure 1). Hispanic patients had approximately 37% greater incidence of diagnosis (incidence rate range, 0.81 [95% CI, 0.77-0.85] to 0.98 [95% CI, 0.93-1.03] diagnoses per 100 person-years; IRR, 1.37; 95% CI, 1.34-1.39). Among Asian or Pacific Islander patients, South Asian patients had the highest diagnosis rates (incidence rate range, 0.99 [95% CI, 0.79-1.18] to 1.38 [95% CI, 1.17-1.59] diagnoses per 100 person-years; IRR, 1.71; 95% CI, 1.65-1.78), followed by East Asian patients (incidence rate range, 0.79 [95% CI, 0.69-0.89] to 1.09 [95% CI, 0.98-1.19] diagnoses per 100 person-years; IRR, 1.47; 95% CI, 1.43-1.51), Southeast Asian patients (incidence rate range, 0.72 [95% CI, 0.65-0.80] to 0.95 [95% CI, 0.86-1.03] diagnoses per 100 person-years; IRR, 1.29; 95% CI, 1.26-1.33), and finally other Asian or Pacific Islander patients (incidence rate range, 0.50 [95% CI, 0.43-0.57] to 0.79 [95% CI, 0.73-0.84] diagnoses per 100 person-years; IRR, 0.98; 95% CI: 0.96-1.01) (Figure 1 and Figure 2). Annual age-standardized incidence rates and confidence intervals are listed in eTable 5 in Supplement 1.

Finally, we assessed the demographic characteristics of patients who received a diagnosis of fibroids in each racial and ethnic group (Table 2). For comparison, the demographic characteristics of the overall population at risk in 2009 and 2022 are shown in eTable 6 in Supplement 1. South Asian patients were more likely than other groups to be younger than 35 years at diagnosis, which does not appear to be explained by the age distribution of the group at risk. For example, among South Asian patients in 2022, 9456 of 25 598 patients without a fibroid diagnosis (37%) and 106 of 318 patients with a fibroid diagnosis (33%) were younger than 35 years (compared with 101 016 of 301 840 patients without a diagnosis [34%] and 284 of 1851 patients with a diagnosis [15%] for White patients, 19 803 of 50 621patients without a diagnosis [39%] and 198 of 938 patients with a diagnosis [21%] for Black patients, and 88 704 of 210 170 patients without a diagnosis [42%] and 301 of 1671 patients with a diagnosis [18%] for Hispanic patients). At diagnosis, East Asian, South Asian, and other Asian or Pacific Islander patients were also more likely to live in the least-deprived quartile of neighborhoods, and all Asian ethnic subgroups were more likely than White, Black, and Hispanic patients to have a BMI less than 25; this pattern of neighborhood residence and BMI was also observed among the patients in each group who did not receive a fibroid diagnosis. Finally, East Asian, Southeast Asian, South Asian, and other Asian or Pacific Islander patients also were less likely than other racial and ethnic groups to have *ICD-9* or *ICD-10* coded symptoms in the 2 years before diagnosis. Among Asian subgroups, having no symptoms ranged from 1203 of 2983 other Asian or Pacific Islander patients (40%) to 2855 of 5997 East Asian patients (48%), while 9062 of 26 992 White patients (34%), 4479 of 13 405 Black patients (33%), and 5358 of 19 236 Hispanic patients (28%) had no symptoms (Table 2). Similarly, Asian or Pacific Islander patients were somewhat less likely than other groups to have each of the specific symptoms examined (bleeding, dysmenorrhea, dyspareunia, lower abdominal or pelvic pain, and/or urinary incontinence) at diagnosis and less likely to have received their first diagnosis in the emergency department (Table 2).

**Table 2.** Demographic and Clinical Characteristics at Time of Fibroid Diagnosis by Race and Ethnicity, Among Patients With a Fibroid Diagnosis

Characteristic	Participants, No. (%) (N = 84 206)							*P* value
	Black (n = 13 405)	East Asian (n = 5997)^a^	Hispanic (n = 19 236)	Southeast Asian (n = 6903)^b^	South Asian (n = 2983)^c^	Other Asian or Pacific Islander (n = 5379)^d^	White (n = 26 992)	
Overall diagnosis rate per 100 person-years (95% CI), 2009-2022	2.27 (2.23-2.31)	1.18 (1.14-1.21)	1.04 (1.02-1.06)	1.01 (0.99-1.04)	1.33 (1.27-1.40)	0.75 (0.73-0.77)	0.77 (0.76-0.78)	
Age, y								
18-29	958 (7)	168 (3)	1105 (6)	233 (3)	300 (10)	253 (5)	1046 (4)	<.001
30-34	2001 (15)	869 (14)	2377 (12)	888 (13)	790 (26)	769 (14)	3037 (11)	
35-39	2763 (21)	1171 (20)	3610 (19)	1411 (20)	630 (21)	1020 (19)	4563 (17)	
40-44	3231 (24)	1299 (22)	4912 (26)	1778 (26)	574 (19)	1268 (24)	5944 (22)	
45-49	2953 (22)	1585 (26)	5005 (26)	1798 (26)	530 (18)	1397 (26)	7405 (27)	
50-54	1499 (11)	905 (15)	2227 (12)	795 (12)	159 (5)	672 (12)	4997 (19)	
Neighborhood Deprivation Index quartile^e^								
1: Least deprived	1110 (8)	1978 (33)	1888 (10)	1001 (15)	1305 (44)	1615 (30)	6496 (24)	<.001
2	3096 (23)	1972 (33)	4758 (25)	2192 (32)	911 (31)	1604 (30)	9841 (36)	
3	4095 (31)	1449 (24)	6151 (32)	2343 (34)	529 (18)	1342 (25)	7287 (27)	
4: Most deprived	5084 (38)	592 (10)	6428 (33)	1359 (20)	235 (8)	800 (15)	3336 (12)	
Body mass index^f^								
<25.0	1980 (15)	3546 (59)	3367 (18)	2884 (42)	1160 (39)	2174 (40)	8356 (31)	<.001
25.0 to <30.0	3132 (23)	1217 (20)	5916 (31)	2123 (31)	999 (33)	1259 (23)	7062 (26)	
30.0 to <35.0	2903 (22)	314 (5)	4281 (22)	814 (12)	355 (12)	551 (10)	4363 (16)	
≥35	3939 (29)	101 (2)	3673 (19)	333 (5)	123 (4)	319 (6)	4126 (15)	
Unknown or missing	1451 (11)	819 (14)	1999 (10)	749 (11)	346 (12)	1076 (20)	3085 (11)	
Symptoms								
None recorded	4479 (33)	2855 (48)	5358 (28)	2813 (41)	1203 (40)	2263 (42)	9062 (34)	<.001
Bleeding	6198 (46)	2190 (37)	9572 (50)	2885 (42)	1199 (40)	2097 (39)	13015 (48)	<.001
Dysmenorrhea	1737 (13)	392 (7)	2032 (11)	579 (8)	290 (10)	412 (8)	2515 (9)	<.001
Dyspareunia	204 (2)	75 (1)	513 (3)	96 (1)	47 (2)	85 (2)	599 (2)	<.001
Lower abdominal or pelvic pain	3918 (29)	1155 (19)	6736 (35)	1653 (24)	779 (26)	1287 (24)	7160 (27)	<.001
Urinary incontinence	489 (4)	170 (3)	1277 (7)	186 (3)	85 (3)	166 (3)	1297 (5)	<.001
Received diagnosis in emergency department	1684 (13)	314 (5)	2650 (14)	776 (11)	326 (11)	411 (8)	2893 (11)	<.001

^a^ East Asian included Chinese, Japanese, and Korean.

^b^ South Asian included Asian Indian, Bangladeshi, East Indian, Nepali, Sri Lankan, and any South Asian ethnicity not otherwise specified.

^c^ Southeast Asian included Filipino, Vietnamese, and other Southeast Asian (ie, any Southeast Asian ethnicity not otherwise specified).

^d^ Other Asian or Pacific Islander includes Native Hawaiian or Pacific Islander, multiethnic Asian, and other or unspecified Asian (ie, any other Asian ethnicity not otherwise specified).

^e^ In total, 98 participants were missing Neighborhood Deprivation Index values at diagnosis; numbers not shown due to small cell sizes.

^f^ Body mass index was calculated as weight in kilograms divided by height in meters squared.

## Discussion

In this cohort study conducted over a 14-year period in a large and racially diverse integrated health care delivery system, we found that South Asian, East Asian, and Southeast Asian patients had 71%, 47%, and 29% higher age-standardized fibroid diagnosis rates, respectively, than White patients. We confirmed that Black patients had more than 3 times the fibroid diagnosis rate of White patients (consistent with prior US research)^19,20^ and reported that Hispanic patients had 37% higher incidence of fibroid diagnosis compared with White patients. Among patients with a fibroid diagnosis, Asian or Pacific Islander patients had lower BMI and were less likely to have recorded symptoms before diagnosis. Our study is among the first to describe the fibroid diagnosis rate across Asian ethnic subgroups, which suggests that fibroid diagnosis rate may be elevated for Asian individuals compared with White individuals with substantial variability by Asian subgroup. Findings warrant further investigation to characterize contributing factors.^21^

These findings provide new evidence that fibroid diagnosis rate may be greater among Asian patients compared with White patients, which aligns with some but not all previous US studies. For example, evidence from 3 ultrasonography studies^22,23,24^ is inconsistent. One study^22^ of asymptomatic adults found twice the prevalence of fibroids in Chinese participants compared with White participants (22% vs 11%), but 2 studies reported similar prevalence between pregnant White and Asian or Pacific Islander participants^23^ and asymptomatic White, East Asian, and South Asian participants.^24^ However, our findings agree with 2 studies reporting that Asian or Pacific Islander individuals received a diagnosis of fibroids more frequently than White individuals (specifically, a 9% higher diagnosis rate in active-duty military surveillance reports^25^ and a 12% greater prevalence of fibroid diagnosis in a health care system^26^). Notably, among Asian patients, we found that South Asian patients in particular had an elevated rate of fibroid diagnosis compared with White patients. Prior comparative studies on fibroid risk in South Asians have been inconclusive. Differing from our findings, 1 ultrasonography screening study^24^ of asymptomatic individuals reported that 6% of South Asian patients and 8% of White patients had prevalent fibroids, but was limited by small sample size (ie, 18 South Asian participants and 58 White participants). On the other hand, our findings are consistent with preliminary evidence that South Asian genetic ancestry is associated with greater fibroid risk than Northern European ancestry.^27^ Our findings reflect the experience of patients in the US who receive care in an integrated health care system; fibroid diagnosis rates may differ for Asian individuals in other countries or contexts. Further research is needed to understand the potential variation in fibroid burden among Asian ethnicities, given the differences in diagnosis rates observed in our study and growing evidence of variation in numerous health outcomes among Asian subgroups.^16,28^

We found elevated incidence of fibroid diagnosis among Hispanic patients, but most prior literature in US populations reported similar or lower risk of fibroids among Hispanic participants compared with White participants. For example, 2 studies^23,24^ using ultrasonography screening reported lower prevalence of visualized fibroids in Hispanic compared with White participants, while a third study^22^ reported similar prevalence (13% of Hispanic participants and 11% of White participants). In an analysis of electronic health records and in the Nurses Health Study II,^19,26^ Hispanic and White patients had similar prevalence of diagnosed fibroids. In contrast, a surveillance study of active-duty military members reported that Hispanic women received a diagnosis of fibroids at approximately a 20% higher rate than White women.^25^ Additional evidence is needed to clarify the risk of fibroids among Hispanic individuals and to understand the sources of variability in estimates of prevalence and incidence from different study populations.

Our finding that Asian or Pacific Islander patients were less likely than other groups to have *ICD-9* or *ICD-10* coded symptoms at diagnosis aligns with some prior research suggesting that Asian patients may experience or report fibroid symptoms differently. For example, Asian patients may have fewer fibroids; in a recent US ultrasonography screening study^22^ of participants with fibroids, only one-quarter of Chinese participants had multiple fibroids, compared with one-third to one-half of White, Black, and Hispanic patients. Alternatively, cultural differences may influence how symptoms like heavy menstrual bleeding are perceived and reported.^29,30,31^ For example, in a Canadian symptomatic fibroid registry,^29^ East Asian participants had larger fibroids and longer symptom duration than White participants, but reported lower symptom severity. Interestingly, in the same registry,^29^ South Asian patients reported more severe symptoms than White patients. Similarly, Asian patients at the Stanford Fibroid Clinic reported fewer symptoms than White patients, although both groups had similar fibroid sizes and locations.^32^ Further research is needed to investigate how objective (eg, fibroid number and size) and subjective (eg, experience of symptoms) fibroid burden varies by race, ethnicity, and cultural context.

Multiple factors could be underlying the racial and ethnic variation in fibroid diagnosis rates. First, variation in diagnosis rates may reflect true differences in disease incidence or prevalence, including differences in age of initiation, number of fibroids, rate of growth, and the experience of symptoms. True differences in fibroid presentation across racial and ethnic groups may be due to racism and classism manifesting as environmental exposures and stress, rather than genetic variation.^10^ Second, variation in fibroid diagnosis could reflect differences in health care utilization and screening. Specifically, patients receiving infertility care and obstetric ultrasonography, especially at advanced maternal age (which may be racially patterned^33^) may be more likely to receive an incidental fibroid diagnosis. Additionally, it is possible that clinicians more actively screen or diagnose some patients based on assumptions about fibroid risk. Relatedly, our findings may be specific to our reliance on clinical diagnosis as the method of ascertainment. For example, we reported diagnosis incidence rates of 27.7 per 1000 person-years for Black patients, which is similar to the rate of self-reported diagnosis in the Black Women’s Health Study (34.4 per 1000 person-years)^34^ but much lower than the ultrasonography-detected incidence of 53.9 cases per 1000 person-years reported in the Study of Environment, Lifestyle, and Fibroids cohort of younger (23-35 years) Black individuals.^35^ Future studies comparing fibroid incidence across racial and ethnic groups should use ultrasonography for more complete ascertainment. Third, the diagnosis rates in this study could be affected by differences in the distribution of risk factors within each group. For example, all Asian groups had lower BMI, which may facilitate earlier diagnosis with transabdominal ultrasonography.^36^ Future research should examine how health care access and utilization, symptom perception and reporting, and variation in fibroid burden intersect with race and ethnicity to explain racial and ethnic disparities in diagnosis rates.

### Strengths and Limitations

This study had multiple strengths. Importantly, we leveraged data from the KPNC EHR over 14 years, which represented the experiences of a very diverse membership of nearly 2 million patients. Use of the EHR allowed us to provide some of the first information about rates of fibroid diagnosis among South Asian, East Asian, and Southeast Asian individuals. Most previous research combined all Asian ethnic groups into 1 heterogeneous category, concealing this variation; future research should continue to disaggregate heterogeneous ethnic groups, including Hispanic groups. Additionally, we were able to age-standardize incidence rates for each racial and ethnic group to the 2022 US female population, which implies that the reported incidence rates may be broadly representative of diagnosis rates within each group beyond KPNC. The incidence rate of fibroid diagnoses in this study (73-89 cases/1000 person-years) is broadly in line with diagnosis rates in similar studies (eg, 101-140 cases/1000 person-years in a smaller integrated health care system,^26^ 64 cases/1000 person-years among active-duty military members,^20^ and 57-89 claims/1000 person-years in commercial claims data^37^), lending credence to our results. Additionally, we were also able to report clinical and demographic information about the patients receiving a first fibroid diagnosis, including *ICD-9* and *ICD-10* coded symptoms.

This analysis was also subject to limitations. Fibroid diagnosis is an imperfect measure of fibroid prevalence; however, all KPNC members have health insurance, which minimizes variability due to differences in access to screening and symptom evaluation. We could not obtain detailed information about patients’ diagnosed fibroids (eg, location, number, or size), although we reported patients’ recorded symptoms in the 2 years before diagnosis. Additionally, we could not identify the causes for the observed differences in fibroid diagnosis rates among members of Asian ethnicities. Nonetheless, these data provide an initial indication of elevated fibroid diagnosis rates and variation among Asian ethnic groups, deserving further investigation.

## Conclusions

In this cohort study of nearly 2 million KPNC patients, we found that South Asian, East Asian, Southeast Asian, and Hispanic patients had higher age-standardized fibroid diagnosis rates than White patients, and confirmed elevated rates of fibroid diagnosis among Black patients. Further investigation may characterize the factors contributing to the elevated fibroid diagnosis rate among Asian patients compared with White patients. If diagnosis rates reflect true variation in disease prevalence, future research is needed to identify modifiable risk factors and provide appropriate clinical counsel.

## Notes

Supplement 1. **eTable 1.** *ICD* Codes Used to Identify Each Fibroid Symptom **eTable 2.** Demographic Characteristics of Cohort Members Eligible at the Start of Each Year, 2009-2022 **eTable 3.** Number of Censored Participants and Reason for Censoring Each Year, 2009-2022 **eTable 4.** Incidence Rate Ratios and 95% Confidence Intervals for Fibroid Diagnosis Rate by Race and Ethnicity, Comparing Each Year to 2009 **eTable 5.** Annual Incidence rates and 95% Confidence Intervals of Fibroid Diagnosis (Age Adjusted) per 100 Person-Years, by Race and Ethnicity **eTable 6.** Demographic and Clinical Characteristics Comparing Patients With and Without a Fibroid Diagnosis by Race and Ethnicity, 2009 and 2022

Supplement 2.
Data Sharing Statement
