# Supplementary material for: Uterine Fibroid Diagnosis by Race and Ethnicity in an Integrated Health Care System
Source: JAMA Netw Open. 2025 Apr 2;8(4):e255235. doi: 10.1001/jamanetworkopen.2025.5235 (PMC11966321; doi:10.1001/jamanetworkopen.2025.5235)
Supplement: Supplement 1. — eTable 1. ICD Codes Used to Identify Each Fibroid Symptom eTable 2. Demographic Characteristics of Cohort Members Eligible at the Start of Each Year, 2009-2022 eTable 3. Number of Censored Participants and Reason for Censoring Each Year, 2009-2022 eTable 4. Incidence Rate Ratios and 95% Confidence Intervals for Fibroid Diagnosis Rate by Race and Ethnicity, Comparing Each Year to 2009 eTable 5. Annual Incidence rates and 95% Confidence Intervals of Fibroid Diagnosis (Age Adjusted) per 100 Person-Years, by Race and Ethnicity eTable 6. Demographic and Clinical Characteristics Comparing Patients With and Without a Fibroid Diagnosis by Race and Ethnicity, 2009 and 2022 [file jamanetwopen-e255235-s001.pdf]

Supplemental Online Content

Mitro SD, Dyer W, Lee C, et al. Uterine fibroid diagnosis by race and ethnicity in an integrated health care system. *JAMA Netw. Open*. 2025;8(4):e255235. doi:10.1001/jamanetworkopen.2025.5235

eTable 1. *ICD* Codes Used to Identify Each Fibroid Symptom

eTable 2. Demographic Characteristics of Cohort Members Eligible at the Start of Each Year, 2009-2022

eTable 3. Number of Censored Participants and Reason for Censoring Each Year, 2009-2022

eTable 4. Incidence Rate Ratios and 95% Confidence Intervals for Fibroid Diagnosis Rate by Race and Ethnicity, Comparing Each Year to 2009

eTable 5. Annual Incidence rates and 95% Confidence Intervals of Fibroid Diagnosis (Age Adjusted) per 100 Person-Years, by Race and Ethnicity

eTable 6. Demographic and Clinical Characteristics Comparing Patients With and Without a Fibroid Diagnosis by Race and Ethnicity, 2009 and 2022

This supplemental material has been provided by the authors to give readers additional information about their work.

© 2025 Mitro SD et al. *JAMA Network Open*.

eTable 1. *ICD* Codes Used to Identify Each Fibroid Symptom

**Symptom**	**ICD-9**	**ICD-10**
Bleeding	626.2, 626.4, 626.6, 626.8, 626.9, 627.0	N92.0, N92.1, N92.2, N92.3, N92.4, N92.5, N92.6, N93.8, N93.9
Dysmenorrhea	625.3	N94.4, N94.5, N94.6
Dyspareunia	625.0	N94.1*
Lower abdominal or pelvic pain	789.03, 789.04, 789.63, 789.64, 625.9	R10.2, R10.30, R10.31, R10.32, R10.33, R10.813, R10.823, R10.814, R10.824
Urinary incontinence	625.6, 788.30, 788.31, 788.33	N39.3, N39.41, N39.46, R32

© 2025 Mitro SD et al. *JAMA Network Open*.

eTable 2. Demographic Characteristics of Cohort Members Eligible at the Start of Each Year, 2009-2022. Values are column percentages.

	2009		2010		2011		2012		2013		2014		2015	
	Overall	Fibroid Diagnosis	Overall	Fibroid Diagnosis	Overall	Fibroid Diagnosis	Overall	Fibroid Diagnosis	Overall	Fibroid Diagnosis	Overall	Fibroid Diagnosis	Overall	Fibroid Diagnosis
n	734,955	5,846	700,196	5,425	688,957	5,432	699,928	5,271	693,485	5,318	683,255	5,448	718,033	5,629
Age, %														
18-29	29	4	27	5	26	4	26	5	25	5	24	5	23	5
30-34	13	10	14	11	15	11	15	11	16	12	16	13	17	13
35-39	14	18	14	17	14	18	14	17	15	18	15	18	16	19
40-44	14	24	14	25	15	25	15	25	15	25	15	24	15	24
45-49	15	29	15	28	15	28	15	28	14	25	14	25	14	24
50-54	15	15	15	14	16	14	15	14	15	14	15	15	15	14
Race and ethnicity, %														
Black	8	18	7	17	7	18	7	17	7	17	7	16	7	16
East Asian	6	8	6	8	6	8	6	9	6	8	6	7	6	8
Hispanic	20	21	20	21	21	21	21	21	21	22	21	23	22	23
South Asian	2	2	2	2	2	3	2	3	2	4	3	3	3	3
Southeast Asian	8	8	8	9	8	9	8	8	8	9	8	9	8	9
Other Asian / Pacific Islander[a]	5	4	5	3	6	3	6	4	6	5	7	5	8	5
White	45	36	45	36	45	35	45	36	45	34	44	34	43	33
Other Races[b]	2	2	2	2	2	2	2	2	2	2	2	2	2	2
Unknown/Missing	6	2	5	2	4	2	4	1	3	1	3	2	3	2
Parity, %														
0	16	16	19	18	20	20	22	22	23	25	24	25	23	26
1	12	12	13	13	14	14	15	15	16	17	16	16	16	16
2+	26	30	30	35	32	37	35	39	37	39	38	41	38	40
Unknown/Missing	46	42	38	34	34	29	29	24	24	19	23	17	24	19
Body mass index, %														
< 25.0	37	31	37	31	37	32	37	31	37	33	37	32	36	31
25.0 - <30.0	24	28	25	28	25	29	25	28	25	28	25	29	25	28
30.0 - < 35.0	14	17	14	17	14	17	14	17	14	17	14	16	14	17
≥ 35	13	16	13	16	13	16	12	14	12	14	13	15	13	15
Unknown/Missing	12	8	11	8	11	7	12	9	12	9	12	8	13	9
Neighborhood Deprivation Index, %														
Least deprived	15	15	16	16	16	16	16	17	18	18	18	17	17	17
Second quartile	32	30	32	31	32	31	32	32	32	31	32	32	32	32
Third quartile	30	31	30	30	30	31	30	30	28	29	28	30	29	28
Most deprived	23	23	22	23	22	22	21	21	21	22	22	21	22	23
Unknown/Missing	0	0	0	0	0	0	0	0	0	0	0	0	0	0

© 2025 Mitro SD et al. *JAMA Network Open*.

eTable 2. Continued

	2016		2017		2018		2019		2020		2021		2022	
	Overall	Fibroid Diagnosis	Overall	Fibroid Diagnosis	Overall	Fibroid Diagnosis	Overall	Fibroid Diagnosis	Overall	Fibroid Diagnosis	Overall	Fibroid Diagnosis	Overall	Fibroid Diagnosis
n	758,527	6,183	777,101	6,385	810,255	6,729	837,088	6,972	843,519	5,820	862,187	7,012	855,111	6,736
Age, %														
18-29	23	5	22	6	22	5	21	5	21	7	20	5	19	4
30-34	17	14	17	14	18	14	18	15	18	16	18	15	18	15
35-39	16	19	17	19	17	20	17	20	18	19	18	21	18	20
40-44	15	22	15	22	15	23	15	23	15	22	16	23	16	24
45-49	15	26	15	26	15	24	15	25	14	22	14	24	14	24
50-54	15	14	15	14	14	14	14	13	14	13	14	13	15	13
Race and ethnicity, %														
Black	7	16	6	15	6	15	6	15	6	15	6	15	6	14
East Asian	5	8	5	7	5	6	5	6	5	6	5	6	5	6
Hispanic	22	23	23	23	23	23	23	24	24	25	24	24	25	25
South Asian	3	3	3	4	3	4	3	4	3	5	3	5	3	5
Southeast Asian	7	8	7	8	7	8	7	8	7	8	7	7	7	7
Other Asian / Pacific Islander[a]	8	6	9	7	10	7	10	8	11	9	11	11	12	10
White	42	32	41	32	40	32	39	29	38	29	37	28	36	28
Other Races[b]	2	2	2	2	2	2	2	2	2	2	2	2	2	2
Unknown/Missing	4	2	4	2	4	2	5	2	5	2	6	4	6	4
Parity, %														
0	23	26	23	25	23	27	23	26	23	27	21	26	21	26
1	16	17	16	17	16	16	16	16	16	17	15	15	15	15
2+	39	41	39	40	36	37	36	37	35	36	33	33	33	33
Unknown/Missing	22	17	22	17	26	21	26	21	26	20	31	26	31	25
Body mass index, %														
< 25.0	35	30	34	29	33	30	32	28	31	28	25	23	21	20
25.0 - <30.0	25	27	25	28	25	26	24	27	24	28	21	23	18	20
30.0 - < 35.0	14	17	14	17	14	18	14	17	14	18	13	16	12	14
≥ 35	13	16	14	16	14	17	14	17	14	16	13	15	12	14
Unknown/Missing	13	10	13	10	14	10	15	11	17	10	29	23	37	32
Neighborhood Deprivation Index, %														
Least deprived	17	16	19	19	22	22	22	22	23	23	23	25	23	24
Second quartile	32	31	32	32	30	29	29	29	28	28	28	27	28	28
Third quartile	28	28	27	28	28	28	28	28	27	27	27	27	27	28
Most deprived	23	24	21	21	20	21	21	21	21	23	21	21	21	21
Unknown/Missing	0	0	0	0	0	0	0	0	0	0	0	0	0	0

[a]*Other Asian or Pacific Islander includes Native Hawaiian/Pacific Islander, Pacific Islander, multiethnic Asian, other or unspecified Asian.* [b]*Other includes Multiracial, American Indian or Alaska Native.*

© 2025 Mitro SD et al. *JAMA Network Open*.

eTable 3. Number of Censored Participants and Reason for Censoring Each Year, 2009-2022

	**2009**	**2010**	**2011**	**2012**	**2013**	**2014**	**2015**	**2016**	**2017**	**2018**	**2019**	**2020**	**2021**	**2022**
Cohort n	734955	700196	688957	699928	693485	683255	718033	758527	777101	810255	837088	843519	862187	855111
Censoring reason														
Hysterectomy	1356	1339	1305	1385	1427	1365	1477	1509	1501	1497	1579	1283	1686	1912
Age 55	20552	20261	20252	20303	19660	19801	20947	21474	21989	22687	22749	22220	21961	22042
Death	516	449	445	495	444	370	404	415	405	401	425	491	553	531
End enrollment	94621	80207	68609	74022	78874	73792	75695	84931	81961	86462	96643	88487	96743	110468
Diagnosed with fibroid	5846	5425	5432	5271	5318	5448	5629	6183	6385	6729	6972	5820	7012	6736
Not Censored	612064	592515	592914	598452	587762	582479	613881	644015	664860	692479	708720	725218	734232	713422

© 2025 Mitro SD et al. *JAMA Network Open*.

eTable 4. Incidence Rate Ratios and 95% Confidence Intervals for Fibroid Diagnosis Rate by Race and Ethnicity, Comparing Each Year to 2009

	**Black**	**East Asian**	**Hispanic**	**Southeast Asian**	**South Asian**	**Other Asian or Pacific Islander[a]**	**White**
**2009**	Reference	Reference	Reference	Reference	Reference	Reference	Reference
**2010**	0.95 (0.88 - 1.04)	0.93 (0.82 - 1.07)	0.95 (0.88 - 1.03)	0.99 (0.88 - 1.13)	1.15 (0.89 - 1.49)	0.80 (0.66 - 0.97)	0.97 (0.91 - 1.03)
**2011**	1.00 (0.92 - 1.09)	0.98 (0.86 - 1.12)	0.95 (0.88 - 1.03)	1.09 (0.96 - 1.23)	1.20 (0.94 - 1.54)	0.78 (0.64 - 0.95)	0.94 (0.88 - 0.99)
**2012**	0.94 (0.86 - 1.02)	1.02 (0.90 - 1.16)	0.93 (0.86 - 1.01)	0.98 (0.86 - 1.11)	1.08 (0.84 - 1.39)	0.77 (0.64 - 0.94)	0.94 (0.88 - 1.00)
**2013**	0.99 (0.91 - 1.08)	0.96 (0.84 - 1.09)	0.95 (0.88 - 1.03)	1.06 (0.94 - 1.20)	1.39 (1.10 - 1.77)	0.93 (0.78 - 1.12)	0.94 (0.88 - 0.99)
**2014**	0.99 (0.90 - 1.08)	0.95 (0.83 - 1.08)	1.01 (0.94 - 1.10)	1.10 (0.97 - 1.24)	1.14 (0.89 - 1.45)	0.99 (0.83 - 1.17)	1.00 (0.94 - 1.06)
**2015**	1.01 (0.93 - 1.11)	1.02 (0.89 - 1.16)	1.01 (0.94 - 1.09)	1.05 (0.93 - 1.20)	1.17 (0.92 - 1.49)	0.99 (0.83 - 1.17)	0.99 (0.93 - 1.05)
**2016**	1.07 (0.98 - 1.17)	1.07 (0.94 - 1.21)	1.04 (0.97 - 1.12)	1.10 (0.97 - 1.24)	1.15 (0.91 - 1.46)	1.03 (0.88 - 1.22)	1.00 (0.95 - 1.06)
**2017**	0.99 (0.91 - 1.08)	1.05 (0.92 - 1.19)	1.01 (0.94 - 1.08)	1.15 (1.02 - 1.30)	1.38 (1.10 - 1.73)	1.11 (0.95 - 1.30)	1.03 (0.97 - 1.09)
**2018**	1.06 (0.97 - 1.15)	0.95 (0.83 - 1.08)	1.01 (0.94 - 1.08)	1.13 (1.00 - 1.27)	1.50 (1.20 - 1.87)	1.09 (0.93 - 1.27)	1.04 (0.99 - 1.11)
**2019**	1.09 (1.00 - 1.18)	1.00 (0.88 - 1.14)	1.05 (0.98 - 1.13)	1.17 (1.04 - 1.32)	1.42 (1.14 - 1.77)	1.11 (0.96 - 1.30)	0.99 (0.93 - 1.05)
**2020**	0.90 (0.82 - 0.98)	0.76 (0.66 - 0.88)	0.86 (0.80 - 0.93)	0.91 (0.80 - 1.03)	1.21 (0.97 - 1.51)	0.92 (0.78 - 1.07)	0.81 (0.77 - 0.87)
**2021**	1.01 (0.93 - 1.10)	0.95 (0.83 - 1.08)	0.96 (0.90 - 1.03)	1.00 (0.88 - 1.13)	1.51 (1.21 - 1.87)	1.25 (1.08 - 1.45)	0.95 (0.90 - 1.01)
**2022**	0.96 (0.88 - 1.04)	0.99 (0.87 - 1.13)	0.96 (0.89 - 1.03)	1.00 (0.88 - 1.13)	1.49 (1.20 - 1.86)	1.16 (1.00 - 1.34)	0.96 (0.91 - 1.02)

[a]Other Asian or Pacific Islander includes Native Hawaiian/Pacific Islander, Pacific Islander, multiethnic Asian, other or unspecified Asian.

© 2025 Mitro SD et al. *JAMA Network Open*.

eTable 5. Annual Incidence rates and 95% Confidence Intervals of Fibroid Diagnosis (Age Adjusted) per 100 Person-Years, by Race and Ethnicity

	**2009**	**2010**	**2011**	**2012**	**2013**	**2014**	**2015**	**2016**	**2017**	**2018**	**2019**	**2020**	**2021**	**2022**
All patients	0.89 (0.87, 0.91)	0.85 (0.83, 0.87)	0.85 (0.83, 0.87)	0.83 (0.81, 0.85)	0.85 (0.82, 0.87)	0.87 (0.85, 0.90)	0.87 (0.85, 0.89)	0.89 (0.87, 0.92)	0.89 (0.87, 0.92)	0.89 (0.87, 0.91)	0.89 (0.87, 0.91)	0.73 (0.71, 0.75)	0.84 (0.82, 0.86)	0.82 (0.80, 0.84)
Black	2.20 (2.07, 2.33)	2.06 (1.93, 2.19)	2.14 (2.01, 2.27)	2.02 (1.89, 2.15)	2.12 (1.98, 2.26)	2.08 (1.94, 2.22)	2.13 (1.99, 2.26)	2.24 (2.11, 2.38)	2.08 (1.95, 2.21)	2.22 (2.08, 2.35)	2.26 (2.12, 2.40)	1.85 (1.72, 1.97)	2.04 (1.91, 2.16)	1.93 (1.80, 2.05)
East Asian	1.06 (0.96, 1.16)	0.99 (0.90, 1.09)	1.00 (0.90, 1.09)	1.04 (0.94, 1.13)	0.99 (0.89, 1.09)	0.97 (0.87, 1.06)	1.05 (0.95, 1.15)	1.09 (0.98, 1.19)	1.05 (0.95, 1.15)	0.94 (0.85, 1.04)	1.00 (0.90, 1.11)	0.79 (0.69, 0.89)	0.96 (0.85, 1.07)	1.01 (0.89, 1.13)
Hispanic	0.98 (0.93, 1.03)	0.91 (0.86, 0.96)	0.89 (0.84, 0.95)	0.88 (0.83, 0.93)	0.89 (0.84, 0.94)	0.95 (0.90, 1.00)	0.93 (0.89, 0.98)	0.96 (0.91, 1.01)	0.93 (0.89, 0.98)	0.93 (0.88, 0.97)	0.97 (0.92, 1.01)	0.81 (0.77, 0.85)	0.89 (0.85, 0.93)	0.87 (0.83, 0.91)
South Asian	0.99 (0.79, 1.18)	1.12 (0.92, 1.32)	1.23 (1.02, 1.45)	1.11 (0.91, 1.31)	1.38 (1.17, 1.59)	1.06 (0.88, 1.24)	1.12 (0.94, 1.30)	1.01 (0.86, 1.17)	1.26 (1.08, 1.43)	1.36 (1.19, 1.53)	1.30 (1.13, 1.46)	1.15 (0.99, 1.31)	1.30 (1.14, 1.47)	1.27 (1.10, 1.44)
Southeast Asian	0.91 (0.83, 0.99)	0.88 (0.80, 0.96)	0.95 (0.86, 1.03)	0.85 (0.77, 0.92)	0.91 (0.82, 0.99)	0.91 (0.82, 0.99)	0.87 (0.80, 0.95)	0.89 (0.81, 0.97)	0.92 (0.84, 1.00)	0.89 (0.81, 0.97)	0.92 (0.84, 1.00)	0.72 (0.65, 0.80)	0.77 (0.70, 0.84)	0.75 (0.68, 0.83)
Other Asian / Pacific Islander[a]	0.66 (0.57, 0.75)	0.52 (0.44, 0.60)	0.51 (0.43, 0.58)	0.50 (0.43, 0.57)	0.59 (0.52, 0.67)	0.63 (0.55, 0.71)	0.63 (0.56, 0.70)	0.65 (0.58, 0.71)	0.71 (0.64, 0.78)	0.69 (0.63, 0.75)	0.70 (0.65, 0.76)	0.58 (0.53, 0.64)	0.79 (0.73, 0.84)	0.71 (0.66, 0.76)
White	0.69 (0.67, 0.72)	0.66 (0.64, 0.69)	0.65 (0.62, 0.67)	0.65 (0.63, 0.68)	0.65 (0.62, 0.68)	0.69 (0.66, 0.72)	0.68 (0.65, 0.71)	0.68 (0.66, 0.71)	0.70 (0.67, 0.73)	0.71 (0.68, 0.74)	0.67 (0.64, 0.70)	0.56 (0.53, 0.58)	0.63 (0.61, 0.66)	0.64 (0.61, 0.67)
Other Races[b]	0.94 (0.78, 1.10)	0.84 (0.69, 1.00)	0.99 (0.82, 1.16)	0.90 (0.74, 1.07)	0.82 (0.66, 0.98)	0.96 (0.78, 1.14)	0.91 (0.73, 1.08)	1.06 (0.88, 1.25)	0.99 (0.82, 1.16)	0.81 (0.66, 0.97)	1.01 (0.84, 1.18)	0.78 (0.63, 0.93)	0.78 (0.63, 0.93)	0.84 (0.68, 0.99)

[a]*Other Asian or Pacific Islander includes Native Hawaiian/Pacific Islander, Pacific Islander, multiethnic Asian, other or unspecified Asian.* [b]*Other includes Multiracial, American Indian or Alaska Native.*

© 2025 Mitro SD et al. *JAMA Network Open*.

eTable 6. Demographic and Clinical Characteristics Comparing Patients With and Without a Fibroid Diagnosis by Race and Ethnicity, 2009 and 2022

	Black				East Asian				Hispanic			
	2009		2022		2009		2022		2009		2022	
	No dx	Fibroid dx	No dx	Fibroid dx	No dx	Fibroid dx	No dx	Fibroid dx	No dx	Fibroid dx	No dx	Fibroid dx
n	54,315	1,069	50,621	938	40,249	440	38,310	424	147,896	1,212	210,170	1,671
Age, years, %	%	%	%	%	%	%	%	%	%	%	%	%
18-29	31.9	5.2	20.0	6.0	22.3	2.7	8.7	3.1	31.5	5.4	24.1	5.1
30-34	12.5	12.6	19.2	15.1	15.1	9.5	16.4	14.6	15.8	11.3	18.1	12.9
35-39	13.4	20.1	17.5	22.7	16.2	19.1	19.4	20.8	15.4	19.6	16.3	17.4
40-44	13.1	26.8	15.6	24.0	14.7	20.0	19.2	24.5	13.8	25.7	15.2	25.4
45-49	14.3	24.9	13.5	21.2	15.4	31.1	18.3	21.9	12.5	26.7	13.8	26.2
50-54	14.8	10.4	14.3	11.0	16.3	17.5	18.0	15.1	10.9	11.4	12.5	12.9
Parity, %												
0	14.4	15.7	21.4	27.9	18.4	19.1	19.8	32.1	12.0	10.1	18.5	19.3
1	13.9	13.5	17.2	18.2	14.2	11.4	19.9	15.8	12.0	11.2	13.6	13.0
2+	27.1	28.4	34.5	28.9	22.5	26.8	34.9	26.7	34.3	38.6	40.2	45.7
Unknown/Missing	44.6	42.4	26.9	24.9	44.9	42.7	25.4	25.5	41.6	40.0	27.8	22.0
Body Mass Index, %												
< 25.0	20.5	16.6	12.0	11.0	63.8	63.0	38.4	41.7	25.7	20.0	13.2	10.8
25.0 - <30.0	23.9	23.0	16.6	15.4	17.5	24.1	15.5	15.1	28.5	32.3	20.1	23.4
30.0 - < 35.0	18.9	21.0	15.7	18.9	4.4	5.9	4.6	5.4	18.7	22.0	16.4	19.4
≥ 35	25.4	29.5	24.2	25.9	1.8	0.9	1.8	1.7	16.8	18.2	17.4	18.6
Unknown/Missing	11.2	10.0	31.5	28.9	12.5	6.1	39.7	36.1	10.3	7.3	33.0	27.8
Neighborhood Deprivation Index, %												
Least deprived quartile	5.5	5.7	10.6	10.6	27.3	30.0	39.7	41.3	6.9	9.8	11.1	11.7
Second quartile	21.6	25.0	22.2	24.4	35.7	33.9	29.7	28.5	23.3	21.5	21.8	22.5
Third quartile	30.6	30.4	29.1	28.8	24.7	25.0	21.5	21.0	32.0	32.8	31.6	33.8
Most deprived quartile	42.1	38.9	38.0	36.0	11.9	10.9	9.1	9.2	37.7	35.9	35.5	32.1

© 2025 Mitro SD et al. *JAMA Network Open*.

eTable 6. continued.

	South Asian				Southeast Asian				Other Asian / Pacific Islander[a]			
	2009		2022		2009		2022		2009		2022	
	No dx	Fibroid dx	No dx	Fibroid dx	No dx	Fibroid dx	No dx	Fibroid dx	No dx	Fibroid dx	No dx	Fibroid dx
n	12,660	103	25,598	318	54,496	458	55,675	477	37,198	224	99,791	689
Age, years, %	%	%	%	%	%	%	%	%	%	%	%	%
18-29	33.1	10.7	11.4	5.7	27.6	3.7	10.8	1.9	45.7	14.3	42.5	22.3
30-34	23.6	20.4	25.6	27.7	16.0	11.1	16.1	13.2				
35-39	14.8	13.6	26.3	21.4	16.1	17.7	20.1	17.8	17.1	19.6	16.2	19.2
40-44	11.4	21.4	17.8	20.1	13.8	23.4	19.6	28.1	14.6	25.9	14.3	20.2
45-49	9.2	30.1	11.2	19.8	12.9	29.7	17.5	26.4	12.4	29.5	13.9	25.7
50-54	7.9	3.9	7.7	5.3	13.7	14.4	15.9	12.6	10.2	10.7	13.1	12.6
Parity, %												
0	14.5	12.6	12.1	23.6	14.8	17.5	16.4	24.7	13.2	11.2	25.7	28.4
1	18.2	20.4	24.8	25.8	13.7	11.1	17.4	17.8	9.8	10.3	11.8	13.9
2+	24.8	28.2	42.2	30.8	27.2	31.2	42.8	36.3	23.8	28.6	21.3	21.5
Unknown/Missing	42.5	38.8	20.9	19.8	44.3	40.2	23.4	21.2	53.2	50.0	41.2	36.1
Body Mass Index, %												
< 25.0	46.1	41.7	26.2	28.0	46.5	45.6	25.7	30.4	46.8	42.4	29.3	29.6
25.0 - <30.0	29.7	25.2	25.5	25.2	26.9	30.3	21.4	24.7	21.1	28.1	15.9	18.9
30.0 - < 35.0	10.3	16.5	11.2	12.3	10.1	10.3	10.8	9.4	8.2	11.2	7.0	9.0
≥ 35	3.6	2.9	4.3	4.7	4.5	3.9	6.3	5.5	5.6	7.6	4.6	4.6
Unknown/Missing	10.3	13.6	32.8	29.9	12.0	9.8	35.8	30.0	18.3	10.7	43.2	37.9
Neighborhood Deprivation Index, %												
Least deprived quartile	31.2	32.0	47.0	48.4	10.5	13.3	17.3	19.9	19.9	20.1	32.0	37.6
Second quartile	34.2	37.9	25.7	28.3	30.8	28.8	29.7	28.5	30.8	32.1	27.1	26.6
Third quartile	22.2	20.4	19.4	14.8	35.9	38.6	31.9	32.9	28.2	29.9	24.9	23.7
Most deprived quartile	12.1	9.7	7.9	8.5	22.6	19.0	21.1	18.7	20.6	17.9	15.9	12.0

© 2025 Mitro SD et al. *JAMA Network Open*.

eTable 6. Continued.

	White				Other races[b]			
	2009		2022		2009		2022	
	No dx	Fibroid dx	No dx	Fibroid dx	No dx	Fibroid dx	No dx	Fibroid dx
n	324,968	2,111	301,840	1,851	14,760	127	14,288	117
Age, years, %	%	%	%	%	%	%	%	%
18-29	26.7	3.4	15.6	3.3	31.3	3.9	21.6	4.3
30-34	11.5	6.9	17.9	12.0	12.1	7.9	20.5	15.4
35-39	12.6	15.6	18.4	18.7	12.4	15.7	17.3	23.9
40-44	13.9	23.0	16.8	23.2	12.9	26.0	15.7	25.6
45-49	16.9	30.9	14.3	25.2	15.1	29.9	12.5	22.2
50-54	18.2	20.2	17.0	17.6	16.3	16.5	12.4	8.5
Parity, %								
0	18.9	19.1	24.7	29.7	17.2	15.7	23.5	29.9
1	11.5	11.8	15.1	14.0	13.1	9.4	16.0	18.8
2+	24.8	28.1	32.3	32.8	26.9	33.1	33.6	26.5
Unknown/Missing	44.8	41.0	28.0	23.4	42.8	41.7	26.9	24.8
Body Mass Index, %								
< 25.0	38.8	33.7	22.8	21.1	34.7	28.3	20.5	14.5
25.0 - <30.0	24.2	28.1	17.9	19.6	25.2	26.8	18.7	13.7
30.0 - < 35.0	13.5	15.7	11.2	12.9	15.0	16.5	12.7	13.7
≥ 35	13.3	15.2	12.3	14.9	16.1	20.5	14.5	17.9
Unknown/Missing	10.3	7.3	35.8	31.5	8.9	7.9	33.7	40.2
Neighborhood Deprivation Index, %								
Least deprived quartile	18.8	19.1	27.3	28.3	14.8	11.8	24.0	29.9
Second quartile	37.3	37.6	33.3	34.6	31.7	33.1	29.7	28.2
Third quartile	29.2	29.6	25.8	25.3	31.4	33.1	27.0	24.8
Most deprived quartile	14.4	13.6	13.5	11.7	22.0	21.3	19.3	17.1

[a]Other Asian or Pacific Islander includes Native Hawaiian/Pacific Islander, Pacific Islander, multiethnic Asian, other or unspecified Asian. Two age categories for this group were combined due to small sample size. [b]Other includes Multiracial, American Indian or Alaska Native.

© 2025 Mitro SD et al. *JAMA Network Open*.
