# Supplementary material for: Uterine Fibroid Diagnosis by Race and Ethnicity in an Integrated Health Care System
Source: JAMA Netw Open. 2025 Apr 2;8(4):e255235. doi: 10.1001/jamanetworkopen.2025.5235 (PMC11966321; doi:10.1001/jamanetworkopen.2025.5235)
Supplement: Supplement 2. — Data Sharing Statement [file jamanetwopen-e255235-s002.pdf]

Data Sharing Statement

Mitro. Uterine Fibroid Diagnosis by Race and Ethnicity in an Integrated Health Care System. *JAMA Netw Open*. Published April 02, 2025. doi:10.1001/jamanetworkopen.2025.5235

Data
Data available: No
